# Ionic Liquid as Dispersing Agent of LDH-Carbon Nanotubes into a Biodegradable Vinyl Alcohol Polymer

**DOI:** 10.3390/polym12020495

**Published:** 2020-02-24

**Authors:** Valeria Bugatti, Gianluca Viscusi, Antonio Di Bartolomeo, Laura Iemmo, Daniela Clotilde Zampino, Vittoria Vittoria, Giuliana Gorrasi

**Affiliations:** 1 Department of Industrial Engineering, University of Salerno, via Giovanni Paolo II, 132, 84084 Fisciano, Italy; vbugatti@unisa.it (V.B.); gviscusi@unisa.it (G.V.); vvittoria@unisa.it (V.V.); 2 Nice Filler s.r.l., via Loggia dei Pisani, 25, 80133 Napoli, Italy; 3 Physics Department “E.R. Caianiello”, University of Salerno, via Giovanni Paolo II, 132, 84084 Fisciano, Italy; liemmo@unisa.it; 4 Institute for Polymers, Composites and Biomaterials (IPCB)–National Research Council, Via Paolo Gaifami, 18-95126 Catania, Italy; danielaclotilde.zampino@cnr.it

**Keywords:** layered double hydroxides, carbon nanotubes, ionic liquids, electrical properties

## Abstract

A Zn/Al layered double hydroxides (LDHs) hosting carbon nanotubes (80% of CNTs) was synthesized and dispersed into a commercial biodegradable highly amorphous vinyl alcohol polymer at different loading (i.e., 1; 3; 5; 10 wt %). In order to improve the degree of dispersion of the filler into the polymer matrix, an ionic liquid (IL) based on 1-hexadecyl-3-methylimidazolium dimethyl-5-sodiosulfoisophthalate was added to the composites’ mixtures. Structural characterization of filler and polymeric composites was carried out. The analysis of thermal, mechanical and electrical properties of the composites, resulted improved compared to the unfilled material, allowed to hypothesize a good dispersion of the LDH-CNTs lamellar filler into the polymer matrix-assisted by the ionic liquid. This was demonstrated comparing electrical conductivity of composite at 5% of LDH-CNTs in the presence and in the absence of IL. The experimental results showed that the electrical conductivity of the sample with IL is four orders of magnitude higher than the one without IL. Furthermore, the percolation threshold of the whole system resulted very low—0.26% of LDH-CNTs loading, which is 0.21% of CNTs.

## 1. Introduction

Polymers reinforced with different forms of nanostructured carbon, such as fullerenes, graphene, nanoplatelets, nanotubes (CNTs), etc. are currently in increasing investigation. Due to their high aspect ratio and the surface of contact with the matrix, CNTs have definite advantages for the realization of composites with remarkable electrical conductivity and mechanical properties, such as hardness, impact resistance, vibration damping and photo-protectors [1,2,3,4,5]. However, the conductivity obtained so far is below the expectations based on the achievable mixing and high current carrying capability of CNTs. This is mainly due to the CNTs tendency to agglomerate, determining a poor three-dimensional structure of the conductive phase. The key parameters to control for the development of innovative CNTs-based composites with improved mechanical and electrical properties are: (i) The effective dispersion of CNTs (ii) the strong adhesion to the polymeric matrix leading to the formation of strong interfacial bonding. Dispersion is a determinant factor to reach high conductivity, and for this reason, chemical functionalization of CNTs was first tried. To achieve homogeneous dispersion and strong interfacial adhesion between CNTs and polymer matrix, a considerable number of studies have been carried out based on the chemical functionalization of CNTs [6], in which activated organic groups were grafted onto the surface of CNTs. These groups ought to promote the dispersion of CNTs in solvents, as well as in polymer matrix. Moreover, it is expected that the groups also improve the compatibility between CNTs and polymer matrix, resulting in the improvement of the interfacial property between CNTs and polymer matrix [7,8,9,10,11]. The simplest way to obtain an organic group on CNTs is their mild oxidation giving –COOH carbon nanotubes afterwards incorporated into resins. The presence of –COOH chemical groups on the nanotube surfaces was expected to improve the dispersion into the matrix, and therefore, to increase the electrical properties. Conversely, it was found that nanotube functionalization with –COOH results in a remarkable increase of the percolation threshold, as well as in a strong reduction of the conductance of the composite. This behavior was attributed to the fact that the presence of the –COOH groups catalyzes a fraction of homo-polymerization, creating a more adherent coating to the nanotubes that determines a higher tunneling resistance between interacting CNTs, and therefore, a higher percolation threshold and the lower limit value of the conductivity [12]. An alternative approach was the linking of the –COOH group to an anionic clay, zinc- and aluminum-based layered double hydroxide (LDH) [13]. CNTs and LDHs were simply mixed and incorporated into a silicone rubber matrix [14], produced as nano-hybrid from Mg/Al and incorporated into a poly (amide-imide) [15] or self-assembled to generate a three-dimensional CNTs-NiCoAl LDH hybrid by means of a urea-hydrolysis method [16]. Here we use the co-precipitation method to synthesize LDH modified with CNTs-COOH, based on Zn/Al, and its dispersion into a novel material, that is a highly amorphous ethylene-vinyl alcohol copolymer [17]. Different dispersants have been used to avoid CNTs aggregation, in particular solid substances and volatile organic solvents. Recently, ILs have been widely used as effective dispersants of nanofillers, such as CNTs [18,19,20], graphene [21], fullerenes [22], as a compatibilizer in polymer blends [23,24], and to enhance the dispersion of petroleum asfaltene [25]. In this paper, we report the fabrication of polymer composites based on a commercial biodegradable highly amorphous vinyl alcohol polymer and layered double hydroxides hosting carbon nanotubes. In order to improve the degree of dispersion of the filler into the polymer matrix, an ionic liquid, based on 1-hexadecyl-3-methylimidazolium dimethyl-5-sodiosulfoisophthalate was used.

## 2. Materials and Methods

### 2.1. Materials

Zn(NO_3_)_2_·6H_2_O, Al(NO_3_)_3_·9H_2_O, NaOH were purchased from Sigma-Aldrich (Milan, Italy). Carbon nanotubes, CNTs-COOH (3101 Grade), were purchased from Nanocyl S.A. (Sambreville, Belgium) 1-methylimidazole, 1-bromohexadecane, 1,3-dimethyl 5-sulfoisophthalate sodium salt, dichloromethane (DCM), ethyl acetate, tetrahydrofuran (THF), dimethyl sulfoxide-d6 (DMSO-d6), trans-2-[3-(4-tert-butylphenyl)-2-methyl-2-propenylidene]malononitrile (DCTB), were purchased by Sigma-Aldrich (Milan, Italy). All the chemicals were high purity products and were used as received. The polymer is a highly amorphous vinyl alcohol polymer (HAVOH), OKS 8049, kindly supplied from Nichigo G-Polymer (Nippon Gohsei Europe GmbH, Düsseldorf, Germany).

### 2.2. Preparation of ZnAl-CNTs by Co-Precipitation Method

CNTs-COOH were co-precipitated with Zn(NO_3_)_2_ and Al_2_(NO_3_)_3_ giving a hydrotalcite-like solid (LDH), in which carbon nanotubes were linked to the hydrotalcite sheets by ionic bonds. 150 mL of an aqueous solution of Zn(NO_3_)_2_·6H_2_O (2.23 g, 7.5 mmol) and Al(NO_3_)_3_·9H_2_O (1.4 g, 3.75 mmol) were added to 150 mL of a CNTs-COONa solution (3 g, 7.5 mmol) under stirring and under nitrogen flow. The pH slowly reached the value of 9 by adding 1M NaOH. In the end, the precipitate was washed with distilled water and left in the oven at 125 °C for 5 h, under vacuum [26].

Elemental analyses for the detection of Zn and Al atoms were carried out by an atomic absorption spectrophotometer (Model Analyst 100, Perkin Elmer, Milan Italy) using solutions prepared by dissolving the samples in concentrated nitric acid. The C and H atoms were analyzed by an Elemental Analyzer CHNS/O (Model Flash EA 1112, Thermo, Haan Germany), equipped by a thermoconductivity detector. The values of relative percentages and molar ratio of Zn, Al, C and H are reported in Table 1. The Zn/Al molar ratio of the starting solution of the Zn and Al nitrates was confirmed.

The chemical formula obtained from the elemental analysis was the following: [Zn_0.73_Al_0.35_(OH)_2_]C_33.7_ O_8.9_·1.75H_2_O with the value of the molar fraction *x* = *M*_III_ / *M*_III_ + *M*_II_ of 0.32 and molecular weight of 668.57 g/mol; the amount of nanotubes in LDH-CNTs is 80 wt % of the total weight. Therefore, almost all the aluminum is co-precipitated with the zinc ions to obtain a solid with the stoichiometry of two Zn(II) atoms for each Al(III) atom. This corresponds to an ideal arrangement of the brucite-like sheet with each aluminum atom surrounded by six zinc atoms [27].

### 2.3. Preparation of Ionic Liquid (IL)

**Scheme 1:**
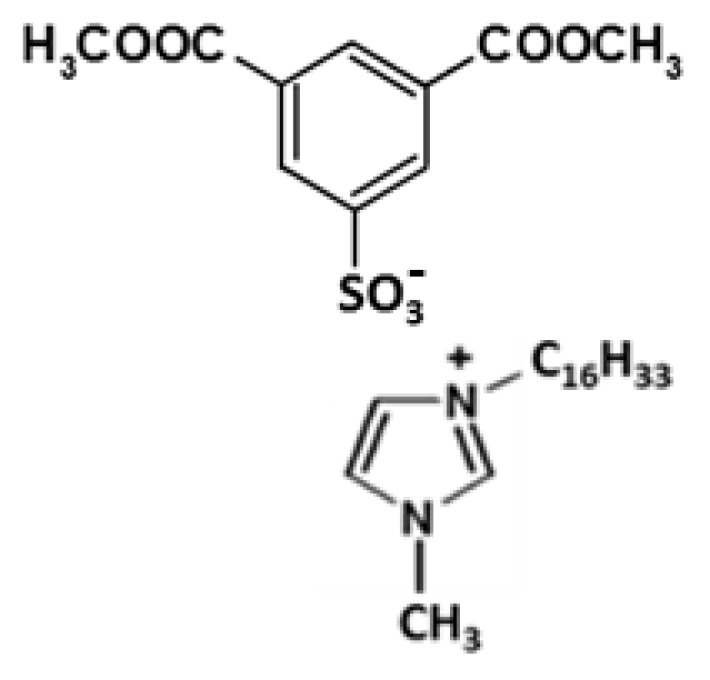
Ionic liquid chemical structure.

The ionic liquid used is 1-hexadecyl-3-methylimidazolium dimethyl-5-sodiosulfoisophthalate (HDmim^+^ DIMSIP^-^) C_30_H_48_N_2_O_7_S. Its chemical structure is shown in Scheme 1.

It was synthesized in two steps. 

The first one involved the alkylation of methyl imidazole with the formation of 1-hexadecyl 3-methyl imidazolium bromide. The second one was a metathesis reaction between the obtained IL bromide and 1,3-dimethyl 5-sulfoisophthalate sodium salt.

#### 2.3.1. 1-Hexadecyl-3-methyl Imidazolium Bromide (Hdmim Br) Synthesis

The synthesis of 1-hexadecyl-3-methyl imidazolium bromide was carried out, and added to an ethyl acetate (4 mL) solution of 1-methylimidazole (1.56 mL, 20 mmol) 1-bromohexadecane (6.4 mL, 20 mmol). The mixture was stirred, under nitrogen, for 24 h at 65 °C. The compound was cooled down to room temperature, obtaining white gelatin, which was filtered and washed several times with ethyl acetate to remove unreacted initial compounds. The white solid obtained was dried under vacuum at 40 °C for 24 h (yield 95.0%).

1H NMR analysis: (200 MHz, DMSO-d6, δ ppm) 0.84 (t, 3H, CH_3_–C_15_), 1.23 (m, 26H, CH_2_), 1.77 (m, 2H, CH_2_–CH_2_–N), 3.85 (s, 3H, CH_3_–N), 4.15 (t, 2H, CH_2_–N), 7.72 (s, 1H, CH in imidazolium ring), 7.78 (s, 1H, CH in imidazolium ring), 9.15 (s, 1H, N–CH–N in imidazolium ring).

#### 2.3.2. 1-Hexadecyl-3-methyl Imidazolium Dimethyl-5-sulfoisophthalate (Hdmim DMSIP) Synthesis

1-hexadecyl-3-methyl imidazolium bromide (20 mmol, 7.77 g) dissolved in 40 mL of dichloromethane (DCM) and a solution of 1,3-dimethyl 5-sulfoisophthalate sodium salt (20.9 mmol, 6.03 g) dissolved in 130 mL of water were put together in a separating funnel and vigorously shaken (30 min) until no precipitate was present in the resulting two-phase mixture. The organic layer was separated, dried over magnesium sulfate, and the residual solvent was removed under reduced pressure. The obtained yellow viscous liquid was washed three times with ethyl acetate and dried under vacuum at 40 °C for 24 h (yield 92.0%). To verify the complete exchange of the bromide counter-ion, a silver nitrate test was performed on an aliquot of the organic layer. A new water solution containing the dimethyl-5sulfoisophthalate sodium salt was exchanged with the organic layer if the exchange was not complete.

1H NMR analysis (200 MHz, DMSO-d6, δ ppm): Signals of imidazolium ring and alkyl chain: 0.84 (t, 3H, CH_3_–C_15_ chain), 1.22 (m, 26 H, CH_2_), 1.75 (m, 2H, CH_2_–CH_2_–N), 3.83 (s, 3H, CH_3_–N), 4.14 (t, 2H, CH_2_–N), 7.69 (s, 1H, CH in imidazolium ring), 7.75 (s, 1H, CH in imidazolium ring), 9.10 (s, 1H, N–CH–N in imidazolium ring). Signals of benzene ring: 3.90 (s, 6H, CH_3_–O), 8.37 (d, 2H, CH, orthoposition with respect to SO_3−_ substituents), 8.42 (d, 1H, CH, paraposition with respect to SO_3−_ substituents).

### 2.4. Preparation of Composites

Composites based on HAVOH with 1% of IL and 1; 3; 5 and 10 wt % of nano-hybrid LDH-CNTs were prepared by dissolving HAVOH in 30 mL of water at 100 °C for 30 min; after this time the powder of LDH-CNTs, in weight ratio (HAVOH: LDH-CNTs) 97:3, 95:5, 93:7 and 90:10 and the IL at 1 wt % were added. The solution containing the nano-hybrid LDH-CNTs, the HAVOH, and IL was then sonicated for 40 min. The mixtures obtained were slowly evaporated in Petri dishes. Films of pure HAVOH and HAVOH/LDH-CNTs/IL for each percentage of nano-hybrid were obtained in the same described experimental conditions. All films, having the same thickness ~300 µm, were dried in a vacuum oven at room temperature for 3 days. In order to better evaluate the effect of the IL on the degree of dispersion of the filler and the resulting physical properties, we prepared a composite based on HAVOH and 5 wt % of LDH-CNTs without IL, using the same experimental conditions described above.

### 2.5. Methods of Investigation

Scanning electron microscopy (SEM) was performed using an EVO MA 10 (Zeiss, Varese Italy) microscope, working in high-vacuum mode.

X-ray diffraction (XRD) patterns were taken, in reflection, with an automatic Bruker diffractometer equipped with a continuous scan attachment and a proportional counter, using nickel-filtered Cu Kα radiation (Kα = 1.54050 Å) and operating at 40 kV and 40 mA, step scan 0.05° of 2*θ* and 3 s of counting time (Karlsruhe, Germany).

Thermogravimetric analyses (TGA) were carried out in air atmosphere with a Mettler TC-10 thermobalance from 30 °C to 800 °C, at a heating rate of 10 °C/min (Mettler-Toledo GmbH, Greifensee, Switzerland).

FTIR was performed using a Bruker spectrometer (Vertex 70, Milan Italy). Spectra were obtained using 32 scans and a 4 cm^−1^.

The elastic modulus, E (Mpa), of the samples was evaluated, in tensile mode, using a dynamometric apparatus INSTRON 4301 (Norwood, MA, USA). Experiments were conducted at room temperature on pure polymer and composites’ films with a deformation rate of 2 mm/min. The specimens were 10 mm wide and ≅500 μm thick. The initial length of the samples was 10 mm. Elastic modulus was derived from the linear part of the stress-strain curves, giving to the samples a deformation of 0.2%. Data were averaged on five samples.

Electrical measurements were performed in a Janis ST-500 cryogenic probe station (Janis Research Company, LLC, 225 Wildwood Avenue, Woburn, MA, USA) connected to a Keithley 4200-SCS semiconductor parameter analyzer (Tektronix, Inc., 14150 SW Karl Braun Drive, P.O. Box 500 Beaverton, OR, USA). Direct current (DC) electrical measurements were performed at atmospheric pressure and room temperature. Films of ~2 × 2 cm^2^ area and ~500–700 µm thickness were sputter coated with two Au pads, on the top and bottom surfaces. The Au pad had a thickness of 200 nm and area around 8 × 8 mm^2^. The DC conductivity along the direction of the film thickness was obtained using a two-probe configuration by measuring the current resulting from the application of a voltage in the range 0–5 V.

## 3. Results

### 3.1. Filler Characterization

**Figure 1:**
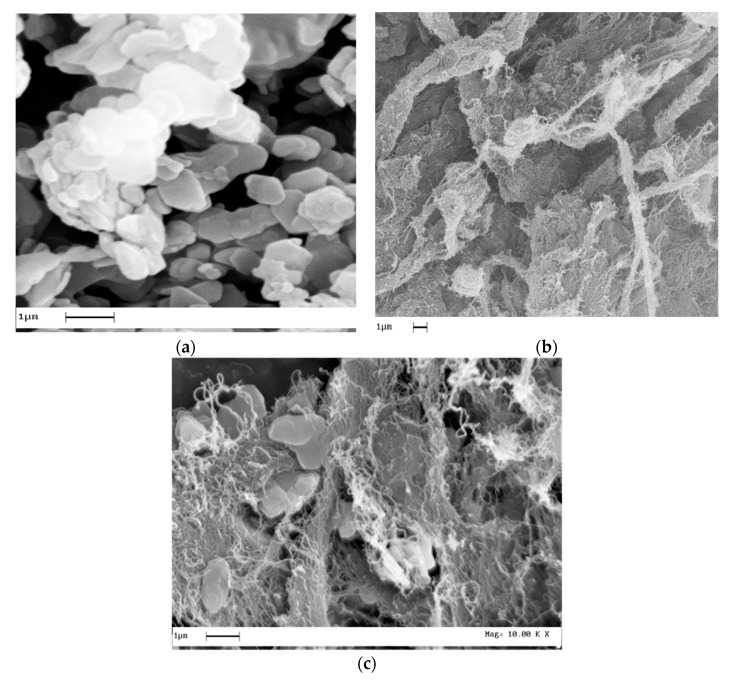
SEM analysis of (**a**) pristine layered double hydroxides (LDH), (**b**) pristine CNT and (**c**) LDH-CNT.

Figure 1 reports the SEM analysis of pristine LDH (a), pristine CNTs (b) and LDH-CNTs (c). The picture of pristine LDH reveals a layered plate-like morphology, typical of such kind of silicate. CNTs show a typical morphology of aggregate nanotubes densely packed, with numerous thinner bundles and single nanotubes coming out of the main bulk. For LDH-CNTs hybrid, it is evident the presence of CNTs among the LDH structure.

**Figure 2:**
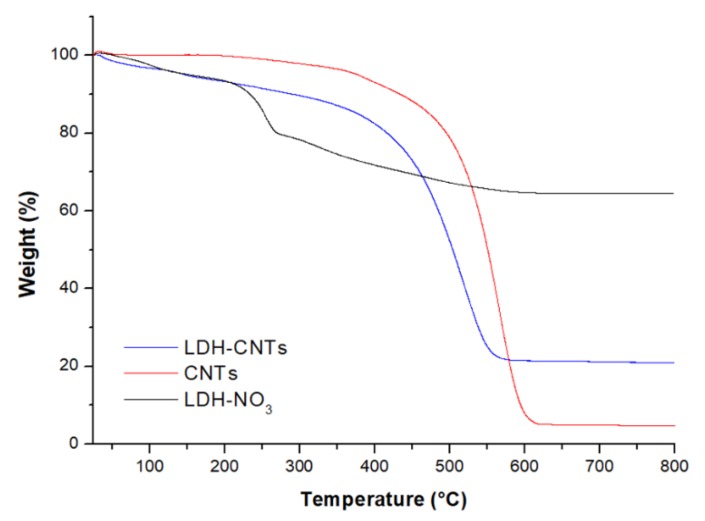
The TGA curves of LDH in nitrate form, the CNTs and the hybrid LDH-CNTs.

Figure 2 shows the TGA curves of the LDH in nitrate form, the CNTs and the hybrid LDH-CNTs. The decomposition temperature of the CNTs is around 550 °C, which indicates that the acid-treated CNTs are thermally stable up to this temperature. LDH-NO_3_ displays three mass-loss steps [28]: The first one, centered at about 108 °C, is due to the loss of physically adsorbed water and water molecules around metal cations, the second one, at about 250 °C, is attributed to the loss of the nitrate anions, and the third one, above 400 °C, due to the de-hydroxylation process. For LDH-CNTs hybrids, three stages of mass loss were also observed in the TG curves. The first mass loss, at approximately 160 °C, is attributed to the removal of loosely bound water molecules from the LDH interlayer. The main mass loss, in the at about 500 °C, is primarily due to the oxidation of CNTs.

**Figure 3:**
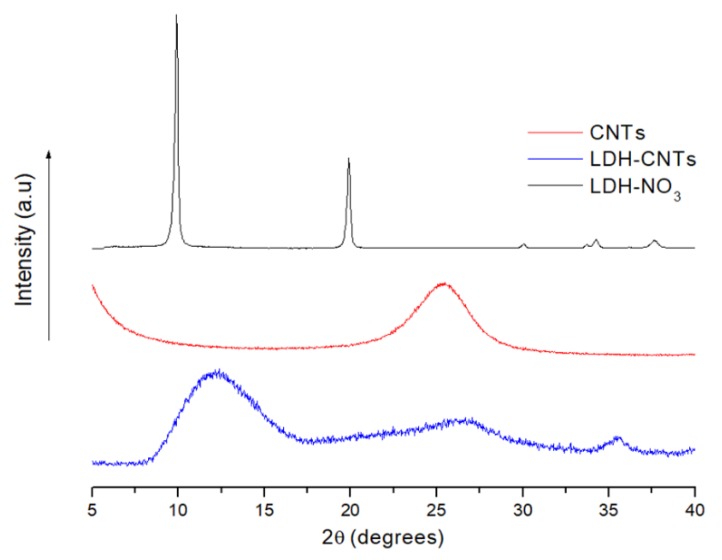
The XRD of LDH in nitrate form, the CNTs and the hybrid LDH-CNTs.

Figure 3 reports the XRD of LDH in nitrate form, the CNTs and the hybrid LDH-CNTs. The LDH-NO_3_ shows the diffraction peaks at 9.8°, 19.8°, 29.8° of 2*θ* corresponding to the basal order reflections of (003), (006), (009), respectively. The peak at 2*θ* = 25.4° for CNTs is typical of (002) basal reflection. The hybrid LDH-CNTs shows peaks of both pristine components, with an evident amorphization of the pristine LDH, after CNTs substitution with nitrate groups.

**Figure 4:**
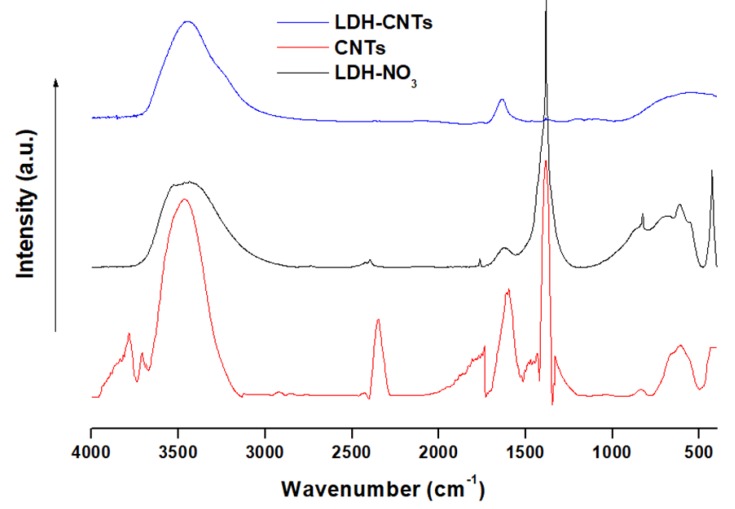
FTIR of LDH in nitrate form, the CNTs and the hybrid LDH-CNTs.

Figure 4 reports the FTIR of LDH in nitrate form, the CNTs, and the hybrid LDH-CNTs. For the LDH-NO_3_ the band at around 3600 cm^−1^ is attributed to the stretching of the –OH bond of the hydroxyl groups and H_2_O molecules. The weak band at 1630 cm^−1^ can be assigned to the H_2_O bending vibration of the interlayer water. The strong peak around 1380 cm^−1^ is attributed to the anti-symmetric stretching mode of the nitrate anion present between the LDH layers. The bands observed around 839 and 670 cm^−1^ can be assigned to the weak out-of-plane symmetric deformation mode and the anti-symmetric deformation mode of nitrate, respectively. The FTIR spectrum of CNTs shows the evidence the absorption band of C=O at around 1600 cm^−1^, and a strong absorption band centered at about 3462 cm^−1^, attributed to the O–H stretching of carboxylic acid moieties from the surface of CNTs. The spectrum of the hybrid LDH-CNTs shows the same peak of C=O and O–H recorded in the pristine fillers, while the absence of the peak at 1380 cm^−1^ confirms the substitution of nitrate anion with the carboxyl groups on the LDHs’ lamellae.

### 3.2. Composites Characterization

**Figure 5:**
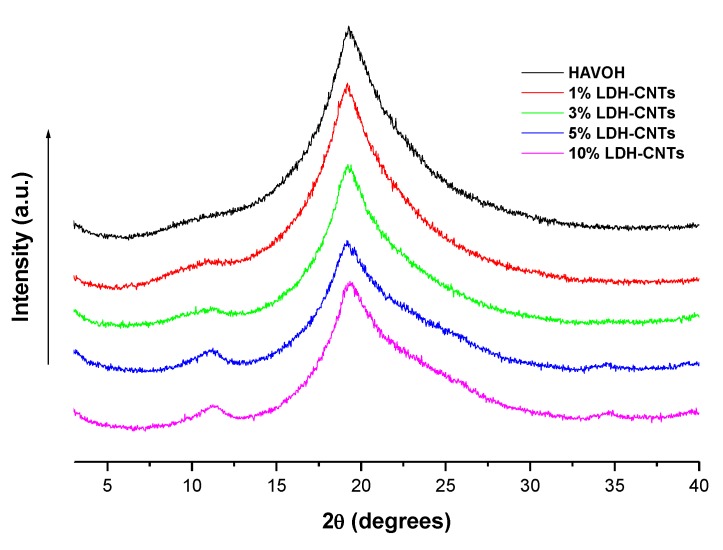
XRD spectra of HVOH and all the composites.

Figure 5 reports the XRD spectra of all the composites. Also, HAVOH with 1% of IL is reported. The amorphous structure of the polymer in composites is retained, also after the mixing with the filler in the presence of ionic liquid. The small peak at 2*θ* = 11.3° is related to the LDH-CNTs.

**Figure 6:**
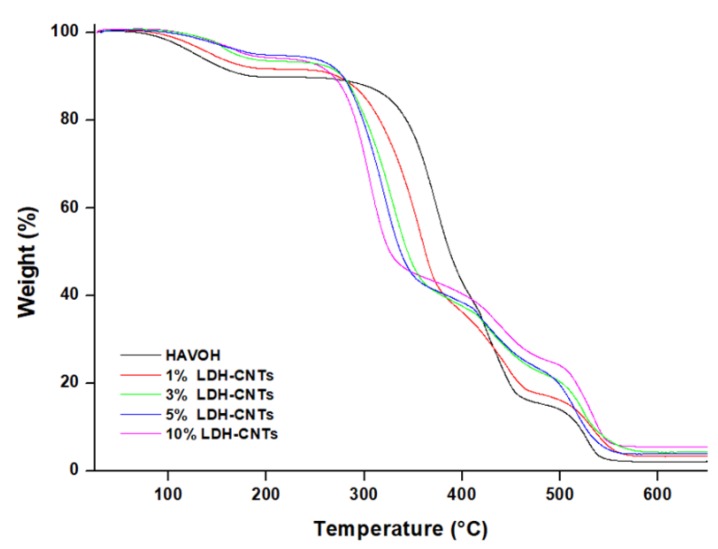
TGA curves for all the composites and the unfilled polymer.

Figure 6 reports the TGA curves for all the composites and the unfilled polymer with 1% of IL. The first decomposition step is due to the loss of water, the second loss in weight is related either to the decomposition of hydroxyl groups or to a split of a part of the polymer chains, a third one is attributed to the decomposition of ethylene segments to carbon chains, and the last one to the volatilization of oxygenated low molecular weight compounds. The introduction of the filler anticipates the second decomposition step and delays the volatilization of low molecular weight compounds. The LDH lamellae represent a barrier to oxygen diffusion into the heated polymer, due to the accumulation of the oxides produced by thermal degradation of the LDH on the surface of the volatizing polymer. The presence of carbon nanotubes, bonded to the lamellae, could also act as physical barriers and/or entrap radicals formed during the decomposition process.

**Figure 7:**
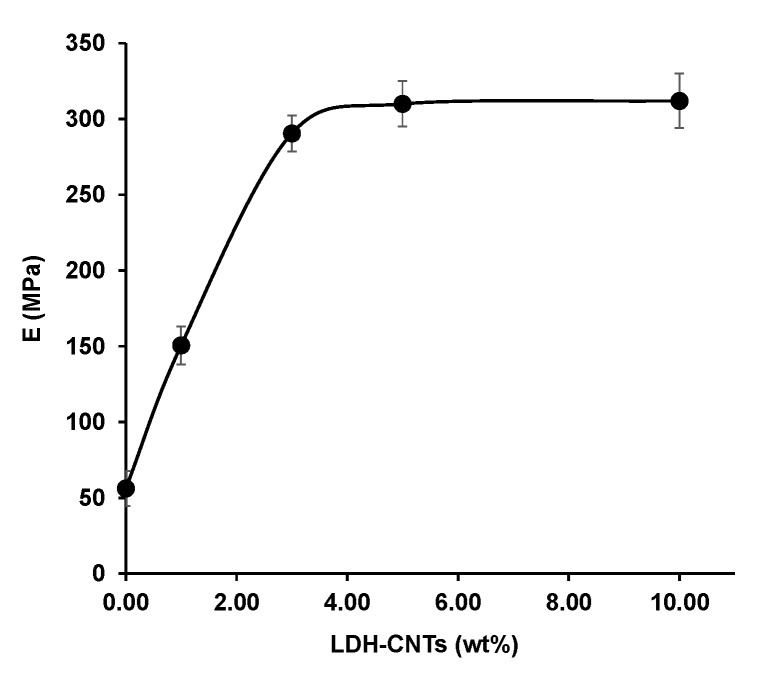
Elastic modulus, E (MPa), as a function of filler loading.

Figure 7 reports the elastic modulus, E (MPa), as a function of filler loading. It increases up to 3% of filler, due to the reinforcing effect of the LDHs lamellae, and reaches a plateau value up to 10% of filler. Being the samples amorphous at any loading composition (see XRD), the reinforcement is only due to the filler dispersion. The degree of load transfer after 5% of filler loading is almost the same for all the composite, resulting in a constant response in mechanical behavior. The sample filled only with 5% of LDH-CNTs display an elastic modulus of 350 ± 20 MPa, higher than the composite filled with 5% of LDH-CNTs and 1% of IL. This is expected because of the plasticizing effect of IL in polymer matrices.

**Figure 8:**
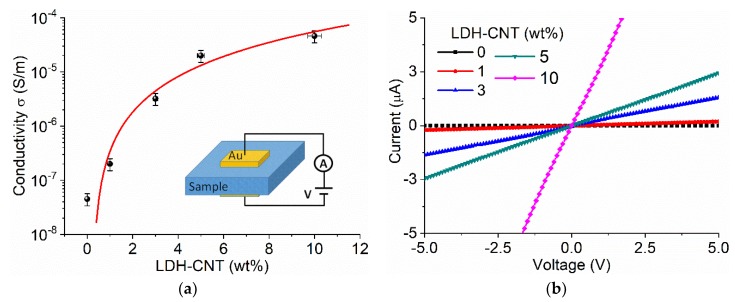
(**a**) Conductivity as a function of the filler loading. The inset shows the sample and measurement setup; (**b**) I-V characteristics for different filler loading.

**Figure 9:**
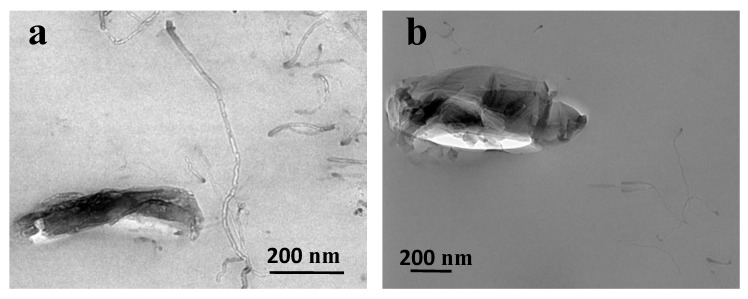
(**a**) SEM of the sample filled with 3 wt % of LDH-CNT and IL and (**b**) SEM of the sample filled with 3 wt % of LDH-CNT without IL.

The DC electrical conductivity σ as a function of the filler loading is shown in Figure 8a. σ is obtained from the I-V curves, shown in Figure 8b, using the formula:

(1) $$\sigma =G\;\left(\frac{t}{A}\right)$$

where *G* = d*I*/d*V* is the sample conductance, that is the slope of the I-V curves of Figure 8b, while *t* and *A* are the sample thickness and Au pad area, respectively. We note that the ohmic behavior of the I-V characteristics, shown in Figure 8b, is typical of CNTs’ filled films and is often found in defective insulators at low bias and high temperature owing to the hopping of carriers between close conducting regions [29,30]. In our sample, hopping can occur between the conductive network LDH-CNTs. The rapid growth of the conductivity with the increasing filler content, up to the maximum $\sigma_{m}=5\times 10^{-5}\frac{S}{m}$ at 10% loading, corresponds to the formation of a conductive pathway (percolation) throughout the sample and is typical of composite materials with well-dispersed and electrically high-conductive fillers. We also measured the electrical conductivity of the sample, based on HAVOH and 3% of LDH-CNTs without IL. The value of electrical conductivity resulted in 2 × 10^−9^ S/m, four orders of magnitude lower than the composite with IL. In order to support such hypothesis we performed SEM analysis on both these samples. Figure 9a reports the SEM image of the sample filled with LDH-CNT and ionic liquid, and Figure 9b the composite without IL. It is evident that the CNTs bonded, and not bonded, to LDH lamellae are better dispersed in the composite with IL (Figure 9a), creating a favored percolation pathway; whereas, in the case of the composite (with no ionic liquid), a separation of CNTs is evident, explaining the lower electrical conductivity.

The lowest concentration of the filler at which the insulating material is converted into a conductive composite is defined as the percolation threshold, which can be estimated from the power-law equation [31]:

(2) $$\sigma =\sigma_{0}{\left(\frac{\eta -\eta_{c}}{1-\eta_{c}}\right)}^{s}$$

where σ and σ_0_ are the DC conductivities of the composite material and the filler, respectively, *η* is the volume fraction of filler loading, *η*_c_ is the weight fraction at percolation (also known as critical weight fraction), and *s* is the power law exponent.

The fit of Equation (2) to the data in Figure 9b (red line) yields *η*_c_ ≈ 0.26%, *σ*_c_ ~ 0.01 S/m and *s* = 2.1. The so-obtained percolation threshold can be considered a very good result when compared with typical values reported in the literature for CNTs based polymer composites [32], and confirms the very good dispersion of the filler into the considered material. It is worth to note that the CNTs amount into the nano-hybrid filler is 80%. This means that the percolation threshold, if referred to the only conductive nanoparticles, is even low.

## 4. Conclusions

The paper reports the preparation of layered double hydroxides (LDHs) hosting carbon nanotubes (80% of CNTs) synthesized by co-precipitation method. The obtained nano-hybrids, characterized by SEM, XRD, FTIR, TGA and elemental analysis, demonstrated the substitution of the nitrate anion with the carboxyl groups on the LDHs’ lamellae. Such nano-hybrid was dispersed into a commercial biodegradable highly amorphous vinyl alcohol polymer at different loading (i.e., 1; 3; 5; 10 wt %). In order to improve the degree of dispersion of the filler into the polymer matrix, an ionic liquid (IL) based on 1-hexadecyl-3-methylimidazolium dimethyl-5-sodiosulfoisophthalate was added to the composites’ mixtures. The structural characterization and the analysis of physical properties of the obtained composites were carried out and compared to the unfilled polymer. The thermal degradation analysis demonstrated that LDH lamellae produce a barrier effect to oxygen diffusion into the heated polymer, due to the accumulation of the oxides produced by thermal degradation of the LDH on the surface of the volatizing polymer. The presence of carbon nanotubes, bonded and adsorbed to the lamellae, could also act as physical barriers and/or entrap radicals formed during the decomposition process. Mechanical properties (i.e., elastic modulus) increased up to 3% of filler, due to the reinforcing effect of the LDH lamellae, and reached a plateau value up to 10% of filler. The electrical properties, evaluated in the range −5–5 V, showed a very low percolation threshold, at 0.26 wt % of nano-filler, that is 0.21 wt % of carbon nanotubes loading, as a probe of the very good dispersion of the filler into the considered biopolymer favored by the IL. The comparison between the electrical conductivity of the sample filled with 5% of LDHs-CNT with IL and without IL showed a difference of four orders of magnitude. The higher electrical conductivity of the composite with IL demonstrated the effect of such additive in favoring a better dispersion of LDH-CNTs into the polymer matrix.

## Figures and Tables

**Table 1:** Values for elemental analysis of ZnAl-CNTs.

	Zn	Al	C	H
**%**	7.2	1.4	60.4	0.82
**Grams**	7.2	1.4	60.4	0.82
**Moles**	0.11	0.05	5.03	0.82
**Molar ratio**	2.1	1.0	96.5	16
